# Effects of postharvest polyamine application and edible coating on maintaining quality of mango (*Mangifera indica* L.) cv. Langra during cold storage

**DOI:** 10.1002/fsn3.802

**Published:** 2019-01-29

**Authors:** Seyed Morteza Zahedi, Marjan Sadat Hosseini, Mahdieh Karimi, Asghar Ebrahimzadeh

**Affiliations:** ^1^ Department of Horticultural Science Faculty of Agriculture University of Maragheh Maragheh Iran; ^2^ Agricultural Biotechnology Research Institute of Iran ‐ Isfahan Branch Agricultural Research, Education and Extension Organization (AREEO) Isfahan Iran; ^3^ Department of Horticultural Sciences Bu‐Ali Sina University Hamedan Iran

**Keywords:** antioxidant activity, ethylene, fungal contamination, phenol, vitamin C

## Abstract

Mango is a tropical fruit which is sensitive to chilling injury. The present work investigated the potential of edible coatings of chitosan and polyamine spermidine in increasing shelf life and quality of mango. The control fruits (treated with distilled water) and the mango fruits treated with different concentrations of chitosan (0.5%, 1.0%, and 2.0%) and spermidine (0.5, 1.0, and 2.0 mM) were studied to improve postharvest characteristics and quality maintenance during cold storage. Parameters such as firmness, weight loss, fungal contamination, total phenol, antioxidant activity, vitamin C, pH, total soluble solids (TSS), titratable acidity (TA), flavor index, color index, and ethylene production were measured after at harvest (0), 8, 16, and 24 days of storage at 15 ± 2°C and relative humidity of 85%–90%. Chitosan and spermidine delayed water loss, firmness, and fungal contamination. Application of chitosan containing ascorbic acid significantly increased phenolic content and antioxidant activity compared to the control plants. It also changed soluble solid content, TA, pH of pulp, and sugar content and decreased ethylene production. The obtained results suggested that chitosan (2%) and spermidine (2 mM) had potential to improve firmness and delay deterioration processes of “Langra” mango after harvest.

## 1 INTRODUCTION

Mango (*Mangifera indica*) is a tropical climacteric fruit whose ripening depends on exogenous or endogenous ethylene. Due to containing an abundant supply of fiber, vitamin C, polyphenols, and carotenoids, mango has superior nutritional value and is entitled as the king of fruits (Nunes, Emond, Brecht, Dea, & Proulx, 2007). Global production of mango reached almost 50 million t in 2016 (FAO, 2016). During ripening, different qualitative and nutritional changes occur in the fruit, for example, changes in color, firmness, accumulation of sugars and organic acids, and also great changes in taste, flavor, aroma, and biochemical materials (Singh, Singh, Sane, & Nath, 2013). Fruit ripening is a complicated process which is complementary to fruit development and acts as the starting point for its senescence. In general, senescence of a fruit happens due to loss of membrane lipids, destabilization of membrane matrix, and lipid peroxidation (Harindra Champa, Gill, Mahajan, & Arora, 2014). Recently, natural active biological products are applied in a large amount for increasing the storage life and quality of fruits and delaying their senescence (Jongsri, Wangsomboondee, Rojsitthisak, & Seraypheap, 2016).

Edible coverings and films increase quality of food products, including fruits, by providing a barrier and a protective structure against the mechanical and oxidative damages, microbial growth, chemical reactions, and gases such as vapor, lipids, and soluble materials (Han & Scanlon, 2005). Chitosan (C_6_H_11_O_4_N_n_) is the second most abundant natural polysaccharide after cellulose and is found in external skeleton of crustaceans, fungal cell walls, and other biological materials (Bourtoom, 2008). This covering improves quality, health, and stability of the physical properties of products by providing a semipermeable barrier to vapor, oxygen, and carbon dioxide between the product and its surrounding atmosphere; it allows only a certain amount of gas to pass through, thus preventing anaerobic respiration and increasing shelf life of the product (Lin, Du, Liang, Wang, & Yang, 2011).

Chien, Sheu, and Yang (2007) and Jitareerat, Paumchai, Kanlayanarat, and Sangchote (2007) reported that chitosan caused delayed weight loss, respiration reduction, ethylene production, and increase of organic acid content and vitamin C in mango. Also, application of chitosan improved storage properties, delayed ripening and senescence, and decreased ethylene activities, fungal infection, and firmness in several climacteric and nonclimacteric fruits, such as jojoba (Wang, Wu, Qin, & Meng, 2014), guava (Hong, Xie, Zhang, Sun, & Gong, 2012), and citrus (Chien et al., 2007).

Polyamines are a group of biomaterials which control ripening of fruits and, due to their aliphatic nitrogen structure, are among the compounds detected in animals, plants, and microorganisms (Mirdehghan & Rahimi, 2016). In plants, there is a competition in production of ethylene and polyamines of spermine, spermidine, and putrescine using the common precursor of S‐adenosyl methionine, yet ethylene and polyamines act oppositely in ripening and senescence processes (Galston & Sawhney, 1990). Application of polyamines had extraordinary effects on the quality of some fruits during storage. Lower weight loss and higher firmness in pomegranate (Mirdehghnan et al., 2007) and grape (Harindra Champa et al., 2014), decreased amount of ACC reductase enzyme in avocado (Li, Parsons, Liu, & Mattoo, 2005), and increased phenol amount in mango (Razzaqa, Khana, Malika, Shahidb, & Ullah, 2014) have been reported for polyamine applications. Malik and Singh (2005) showed that application of polyamines increased postharvest life and vitamin C content and delayed coloring of mango.

Mango is a commercially important fruit and improving its storage life is of special importance. The main objective of this research was to compare the effects of using different concentrations of edible chitosan covering and polyamide spermidine on some properties of mango (Langra cultivar) such as weight loss, firmness, fungal contamination, flavor and taste, as well as some specific compounds such as TSS, phenol content, antioxidant activity, ascorbic acid (vitamin C), and ethylene content during a period of 24 days.

## 2 MATERIALS AND METHODS

### 2.1 Fruit materials

Mango fruits, “Langra” cultivar, were obtained at their commercially ripe (ripe green) stage from a mango garden in Minab (Hormozgan Province, Iran) and were immediately transferred to the laboratory. Healthy and uniform fruits were chosen based on their size, shape, color, and ripening degree and were randomly divided into six groups. After washing with water and drying, their primary visual and chemical properties at harvest time were measured.

### 2.2 Treatment, storage, and conditions of fruit ripening

To prepare chitosan solutions 5, 10, and 20 g of chitosan (Sigma‐ Aldrich Crop, St. Louis, MO, USA) were added to 1 L of 1% acetic acid and 0.5%, 1%, and 2% chitosan solutions were prepared, respectively. After chitosan is completely solved, pH of the solution was fixed at 5 using 1 N sodium hydroxide. Once chitosan solutions were ready, the samples were immersed in the solution for 1 min and then they were dried at room temperature.

Also, treatment solutions were prepared at concentrations of 0, 0.5, 1, and 2 mM spermidine (Sigma‐Aldrich Crop). The samples were treated by immersing in the spermidine solutions for 30 min. An amount of 1 L of distilled water was used for the control treatment. Fruits were stored at 15 ± 2°C and 85%–90% relative humidity during a period of 24 days. The measurements were performed on days 0 (at harvest), 8, 16, and 24.

### 2.3 Quality parameters of the fruits

#### 2.3.1 Changes in weight and firmness of the fruits

In each treatment, mango fruits were weighed by a digital scale with the precision degree of 0.01 before each experiment and also at certain intervals during storage. Weight loss percentage was calculated as follows:

$$\begin{array}{cc} \text{Percentage of loss} & =(\text{initial weight}-\text{secondary weight/initial weight})\\ & \times 100 \end{array}$$

Firmness of the fruit tissue was measured by a penetrometer (OSK‐I‐10576; Ogawa Seiki Co., Tokyo, Japan).

#### 2.3.2 Total antioxidants and total phenol

2, 2‐diphenyl‐1‐picrylhydrazyl (DPPH) assay was utilized to measure total antioxidant activity. An amount of 50 μl of fruit extract was mixed with 1.0 ml of 60 μM DPPH (free radical, 95%; Sigma–Aldrich Chemie GmbH, Steinheim, Germany) in methanol. After being shaken, the mixture was left at 25°C for 30 min and then absorbance of the samples was measured with a spectrophotometer (Cary, 100 Conc, UV‐Visible Spectrophotometer; Varian, USA) (Dokhanieh, Aghdam, Aghdam, & Hassanpour, 2013).

For analysis of the phenolic compound, 100 μl of fruit extract with 400 μl of phosphate buffer and 2.5 ml of Folin reagent (Sigma‐Aldrich) were added to 2 ml of Na_2_CO_3_ (7.5%) and the sample was kept at 50°C for 5 min. The total phenol content was calculated using gallic acid as the standard solution and the results were expressed as mg of gallic acid per 100 g of fresh weight (FW). Absorbance was determined at 760 nm by a digital spectrophotometer (Cary, 100 Conc, UV‐Visible Spectrophotometer; Varian) (Mirdehghan & Rahimi, 2016).

#### 2.3.3 Ascorbic acid (vitamin C) content

Ascorbic acid is a reducing agent and was determined spectrophotometrically against a standard curve using the method provided in O'Grady, Sigge, Caleb, and Opara (2014). Absorbance was determined at 510 nm by a digital spectrophotometer.

#### 2.3.4 Total soluble solids, pH, total acidity (TA), and flavor index

Total soluble solids (TSS) in fruit juice (obtained by homogenizing 30 g of peeled fruit tissue with 90 ml of distilled water for 2 min) were determined in brix degree using a digital refractometer (A.PAL‐1; ATAGO, Tokyo, Japan) at 25°C. pH of the samples was measured with a pH meter (Mettrohm model AG, Switzerland). Also, titratable acidity (TA) of fruit juice was measured by diluting 10 ml of fruit juice in 10 ml of distilled water and then it was titrated with 0.2 N of sodium hydroxide. When the pH value reached 8.4, titration was stopped. Then, percentage of TA was calculated according to the following formula (Roussos, Sefferou, Denaxa, Tsantili, & Stathis, 2011):

TA=[mlNaoH×N(NaOH)×acidmeq.factor/ml juice titrated]×100

The flavor index of the fruit was calculated and reported using the *TSS*/*TA* relation.

#### 2.3.5 Color index

Using a visual experiment, skin color of the mango fruits was evaluated by finding the percentage of the yellow region of fruit skin during storage (Jiang & Joyce, 2000). Fruit skin color was scored based on the following scale: 0 = green, 1 = broken, 2 = below 25% of the fruit color changed, 3 = 25%–50% of the fruit color changed, 4 = 50%–75% of the fruit color changed, and 5 = Full yellow. Color index (CI) was calculated using the following equation: CI = ∑(color scale × relevant fruit)/(the highest scale × total fruit).

#### 2.3.6 Measurement of ethylene production

A volume of 100 g of fresh fruit from each treatment group was placed in a 500 ml glass vessel at 25°C for 1 hr and then, to measure the produced ethylene, 1 ml of the air above the sample was collected and injected into a gas chromatography device (Shimadzo, Japan) equipped with a flame ionization detector. The temperature was kept constant at 80°C and N_2_ was used as the carrier gas (Sayyari, Soleimani Aghdam, Salehi, & Ghanbar, 2016).

#### 2.3.7 Microbial population analysis

Microbial analysis was performed by homogenizing 10 g of the sample from each replicate with 90 ml of sterile peptone water (Oxoid, Basingstoke, UK) for 90 s using a stomacher blender (Bag Mixer 400; InterScience, St.‐Nom‐La‐Bretèche, France). The blend was diluted 10 fold and 1 ml of the final preparation was poured in an agar plate (Mold Count Plates, ABRI, Karaj, Iran) under sterile conditions. All plates were incubated at 25°C for 5 days. The results were expressed as the logarithm of the number of colonies per 10 g of fruit FW (Valverde et al., 2005).

### 2.4 Statistical analysis

The analysis consisted of different levels of treatments and days of storage with three replicates. Statistical analysis of the experimental data was done by the general linear model (GLM) and SAS (version 9.1) software and the comparisons were performed using the Duncan tests at *p* < 0.05.

## 3 RESULTS AND DISCUSSION

### 3.1 Changes in weight and firmness of the fruits

**Figure 1:**
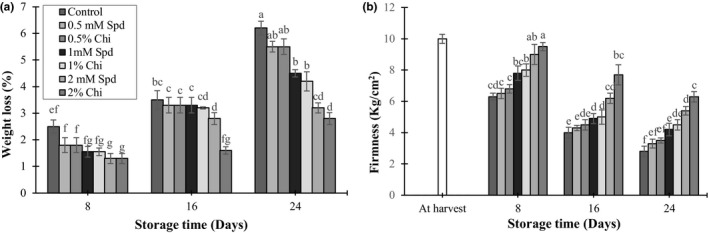
The effect of chitosan coating and spermidine treatments on weight loss (a) and firmness (b) of mango during storage. Each column is the mean of the three replicates and the bars represent the standard error. Values with similar letters are not significantly different (*p* < 0.05)

The effects of both treatments with chitosan and spermidine on weight loss and firmness of the mango fruits were significant (*p* < 0.05). At the end of the storage period, the control fruits had higher weight loss and lower firmness compared to the treated ones (Figure 1a‐b). Treatment with 2% chitosan and 2 mM spermidine delayed weight loss and softening of the fruits, compared to the control treatment. The first mechanism of weight loss in the harvested fresh fruit is vapor diffusion between the internal and external phases, which ultimately results in increased transpiration and weight loss of the fruit (Suseno, Savitri, Sapei, & Padmawijaya, 2014). The positive effect of chitosan on preventing weight loss has also been reported in strawberry (Hernandez‐Munoz, Almenar, Valle, Velez, & Gavara, 2008), guava (Hong et al., 2012), and mushroom (Jiang, Feng, & Li, 2012).

Also, application of spermidine had effects on delaying weight loss in fruits. The effects of polyamines on preventing weight loss have been reported in mango (Razzaqa et al., 2014), tomato (Li et al., 2005), and lemon (Valero, Martinez‐Romero, Serrano, & Riquleme, 1998). Most of the climacteric fruits show weight loss because of water evaporation from the skin surface of the fruit during storage (Sharma, Singh, & Goswami, 2001). Malik and Singh (2005) expressed that weight loss of the fruits treated by polyamines was due to their significant effect on decreasing their respiration. Firmness of mango tissue decreased during storage. Firmness of fruit tissue is related to the structure of and compounds between cell walls. Changes in the structure of cell walls including decreases in hemicellulose and galactose, dissolution of pectines, activities of hydrolyzing enzymes such as polygalactronase, and rapid production of ROS soften fruit tissue during ripening and senescence (Cheng et al., 2008). Previous studies have reported preventing decrease in fruit firmness with chitosan for mango (Jongsri et al., 2016), as well as papaya (Ali, Muhammad, Sijam, & Siddiqui, 2011), guava (Hong et al., 2012), and grape (Al‐Qurashi & Awad, 2015) and with a combination of chitosan and aloe vera gel for papaya and cherry (Ali et al., 2011; Martinez‐Romero et al., 2006). Some hormones and chemicals such as polyamines can decrease senescence and also fruit tissue softening (Valero, Martinez‐Romero, & Serrano, 2002). Polyamines can prevent fracture of the pectin groups by attaching to them and therefore, delay fruit softening (Saftner & Baldi, 2007).

### 3.2 Antioxidant activity and phenol contents

**Figure 2:**
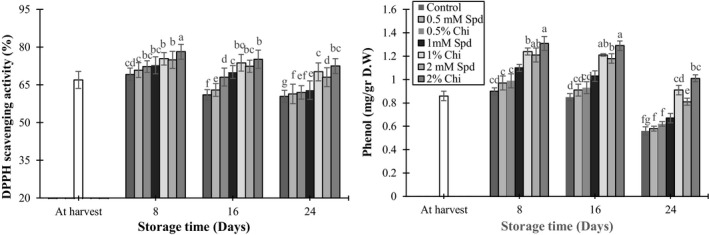
The effect of chitosan coating and spermidine treatments on total antioxidant activity (a) and phenol (b) of mango during storage. Each column is the mean of the three replicates, and the bars represent the standard error. Values with similar letters are not significantly different (*p* < 0.05)

The effect of different concentrations of spermidine and chitosan solutions on total antioxidant and phenol contents of the mango fruits was significant (*p* < 0.05). According to Figure 2a, total antioxidant content of the mango fruits decreased by increasing the storage period. At the end of the storage period, fruits treated with 1% and 2% chitosan solutions had higher phenol content and antioxidant activity compared to the control fruits and other treatments (Figure 2a–b). It has been reported that chitosan solution increased the potential of scavenging reactive oxygen species (ROS) which led to increased content of phenol and antioxidant in mango and table grape fruits (Jongsri et al., 2016; Meng, Li, Liu, & Tian, 2008). At the time of fruit ripening, production of ROS increased while antioxidative defense system decreased (Kim, Brecht, & Talcott, 2007). Also, in tomato (Liu, Tian, Meng, & Xu, 2007), cherry (Dang et al., 2010), orange (Zeng, Deng, Ming, & Deng, 2010), and guava (Hong et al., 2012), treatment with different concentrations of chitosan induced activation of antioxidant enzymes of CAT, SOD, and POD which are responsible for a major part of the antioxidant potential during storage.

### 3.3 Ascorbic acid (vitamin C) content

**Figure 3:**
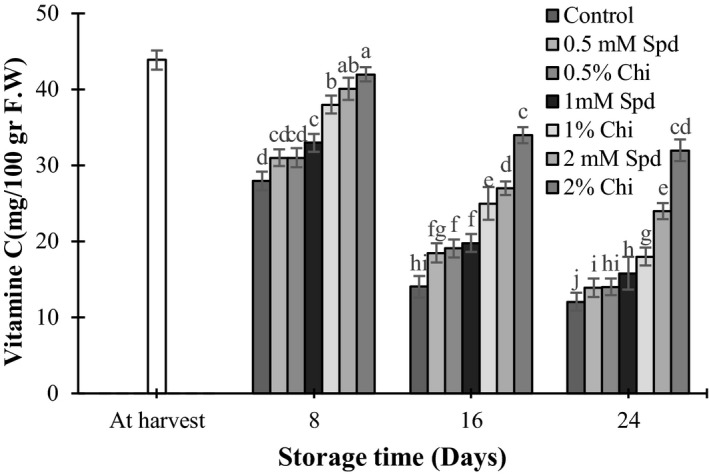
The effect of chitosan coating and spermidine treatments on ascorbic acid of mango during storage. Each column is the mean of the three replicates and the bars represent the standard error. Values with similar letters are not significantly different (*p* < 0.05)

Treatment with different concentrations of chitosan and spermidine had significant effects on ascorbic acid content of the mango fruits (*p* < 0.05). The results showed that by increasing the storage period, ascorbic acid content of the fruits decreased; it was lower in control treatments than the other ones. Fruits treated with 2% chitosan had the highest ascorbic acid content (Figure 3). During storage and fruit ripening, vitamin C content of the fruits decreased quickly due to ascorbinase (the enzyme that oxidizes and decomposes ascorbic acid) activity. Coating fruits with coverings such as chitosan increased cytochrome oxidase activity by decreasing the internal oxygen content of the fruit and this enzyme can significantly decrease decomposition rate of ascorbic acid (Ozden & Bayindirli, 2002). Similar results were reported for different cultivars of mango such as Nam Dok Mai and Kent (Cisse, Polidori, Montet, Loiseau, & Ducamp‐Collin, 2015; Jongsri et al., 2016), carambola fruit (*Averrhoa carambola* L.) (Gol, Chaudhari, & Rao, 2015), and guava (*Psidium guajava* L.) (Hong et al., 2012) which were treated by 1% or 2% chitosan.

### 3.4 TSS, TA, flavor index, and pH

**Figure 4:**
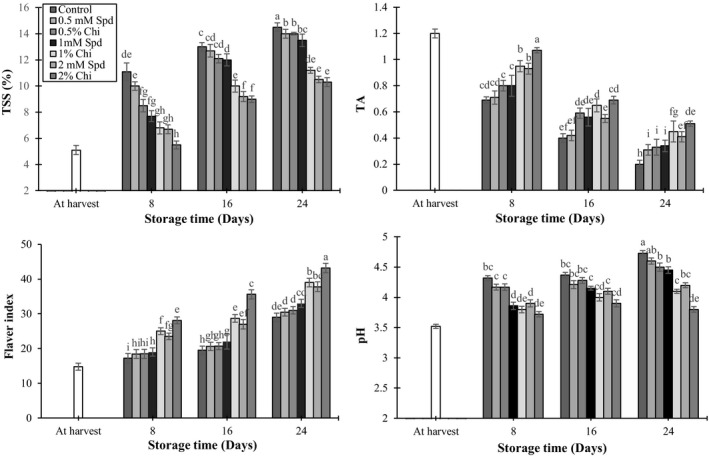
The effect of chitosan coating and spermidine treatments on total soluble solids (a), titrable acidity (b), flavor index (c), and pH (d) of mango during storage. Each column is the mean of the three replicates, and the bars represent the standard error. Values with similar letters are not significantly different (*p* < 0.05)

As can be seen in Figure 4a–d, treatment with chitosan and spermidine had significant effects on the amount of TSS, total acidity, flavor index, and pH of mango (*p* < 0.05). TSS increased during storage and TSS of the control fruits was higher than the other treatment groups. Increase in TSS was slower in groups treated with 2% chitosan and 2 mM spermidine (Figure 4a). Because of ripening of fruits during storage and accumulation of soluble carbohydrates, increase in TSS content of the fruit juice seems logical. Treatment of mango fruits with high concentrations of chitosan had a significant effect on TSS reduction (Jongsri et al., 2016). In both groups treated by chitosan and spermidine and the control group, the acid content of the fruits decreased (Figure 4b). Decline in acid content was fast in control fruits and slow in fruits treated with chitosan and spermidine while the highest acid content was observed in the 2% chitosan treatment. Decrease of TSS and increase of TA in mango fruits were directly related to the decrease of ethylene production and respiration (Jongsri et al., 2016) and this process in fruits treated by chitosan and polyamine was also reported by Khan, Singh, Abbasi, and Swinny (2008) and Hong et al. (2012). Furthermore, increase in flavor index was slower in the control fruits but faster in the fruits treated by 2% chitosan and then 1% chitosan and 2 mM spermidine (Figure 4c).

In chitosan and spermidine treatments and also in the control treatment, pH values of the fruits increased gradually during storage. But increase in pH of the fruits treated with 1% and 2% chitosan and 2 mM spermidine was lower than the other treatments at the end of the storage period (Figure 4d). It seemed that increase in acid content in treatments such as spermidine may result from the active role of these substances in inhibiting storage stress. Also by decreasing tissue respiration, consumption of organic acids decreased during storage which resulted in an increase in acid and a decrease in pH (Leiting & Wicker, 1997). Wang, Wang, Jiang, and Zhao (2007) considered that combined application of chitosan and polyphenols in mango is the reason for a decrease in pH and increase in acid content of the fruit juice.

### 3.5 Color index

**Figure 5:**
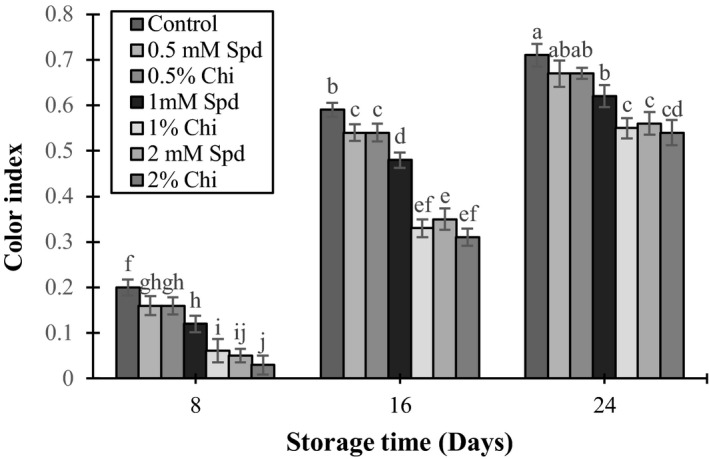
The effect of chitosan coating and spermidine treatments on skin coloration of mango during storage. Each column is the mean of the three replicates, and the bars represent the standard error. Values with similar letters are not significantly different (*p* < 0.05)

The effect of different concentrations of spermidine and chitosan solutions on color change of the fruit was significant (*p* < 0.05) (Figure 5). As can be seen in Figure 5, start of fruit ripening (day 16) and increase in storage duration increased color change in the fruits. Among all treatments done on different days, the amount of color change in mango fruits of the control treatment was higher than that of the other groups and by increasing the concentration of spermidine and chitosan solutions, the yellow color of the fruit skin reduced. The treatment with 1% and 2% chitosan and 2 mM spermidine had the highest effect on decreasing fruit color change.

One of the ripening indexes of mango is its skin color change. Color of a fruit is a very important factor in evaluating its quality. Delayed color change due to using chitosan was reported for a lot of fruits including mango (Zhu, Wang, Cao, & Jiang, 2008), guava (Hong et al., 2012), and plum (Liu, Yuan, Chen, Li, & Liu, 2014). Ali et al. (2011) reported that there was an increase in the amount of CO_2_ inside the fruit as well as a decrease in production of ethylene due to coating fruits with chitosan, which was followed by decreased respiration rate and color change (Martinez‐Romero et al., 2006). Polyamines decrease hydraulic activity of the tilacoide membrane enzymes (Lester, 2000) and the reports showed that polyamines delayed decomposition of chlorophyll and production of carotenoids as well as color changes of fruit skin during storage (Malik & Singh, 2005; Martinez‐Romero, Serrano, Carbonell, Burgos, & Valero, 2002; Valero et al., 2002).

### 3.6 Measurement of ethylene production

**Figure 6:**
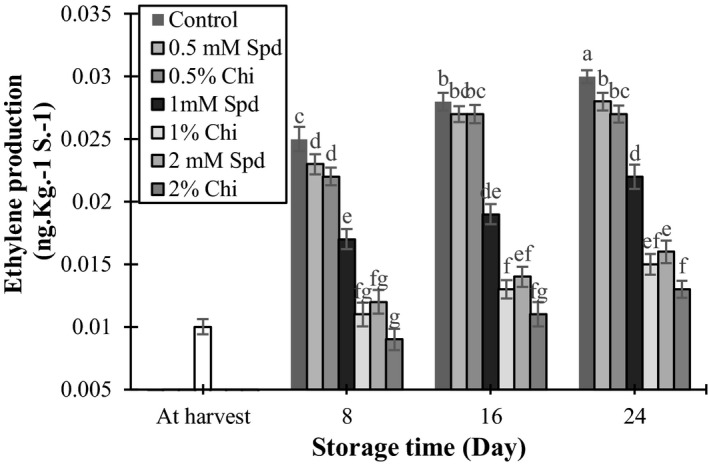
The effect of chitosan coating and spermidine treatments on ethylene production of mango during storage. Each column is the mean of the three replicates, and the bars represent the standard error. Values with similar letters are not significantly different (*p* < 0.05)

Figure 6 shows changes in ethylene production rate in mango fruits during storage at 15 ± 2°C for 24 days. Ethylene production rate was significantly (*p* < 0.05) lower in mango fruits treated with spermidine and chitosan on days 8, 16, and 24. At all measuring times, ethylene production was lower in the 2% chitosan treatment compared with the other treatments. The treatment with 1% chitosan and 2 mM spermidine had the second lowest ethylene production. These results showed that chitosan efficiently decreased ethylene production in mango fruits. Previous studies have shown that chitosan, like a barrier film, provides a selective membrane for permeation of ethylene into or out of the fruit which ultimately decreases ethylene production by the fruit (Ali et al., 2011). Similar results were reported for mango (Jitareerat et al., 2007; Jongsri et al., 2016), papaya (Ali et al., 2011), grape (Romanazzi, Lichter, Mlikota Gabler, & Smilanick, 2012), and litchi (Dong, Cheng, Tan, Zheng, & Jiang, 2004). Polyamines showed contradictory effects, as increase in the content of one of them resulted in decrease in the amount of the other one in the fruit (Valero et al., 2002). Polyamines such as spermidine and putrescine delayed fruit ripening by decreasing respiration (Perez Vicente et al., 2002) and ethylene production (Barman, Ram, & Pal, 2011; Serrano, Martinez‐Romero, Guillen, & Valero, 2003; Zokaee‐Khosroshahi, Esna‐Ashari, & Ershadi, 2007).

### 3.7 Measurement of microbial activity

**Figure 7:**
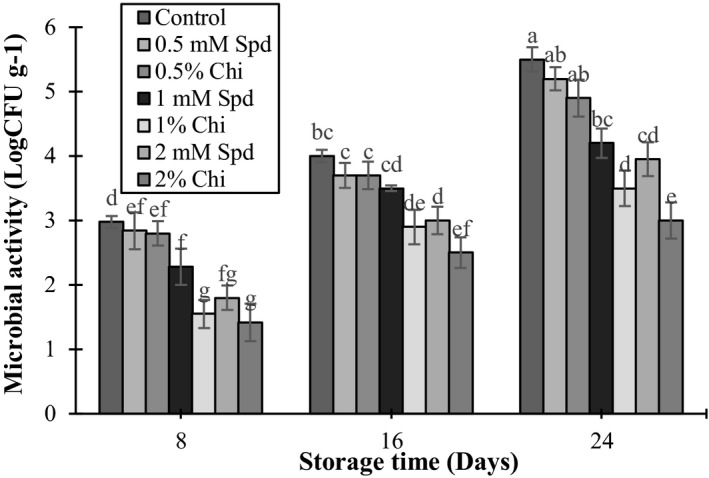
The effect of chitosan coating and spermidine treatments on microbial activity of mango during storage. Each column is the mean of the three replicates, and the bars represent the standard error. Values with similar letters are not significantly different (*p* < 0.05)

Postharvest application of chitosan and spermidine decreased the microbial activity during storage. At the end of the storage period (day 24), treatments with 1% and 2% chitosan and 2 mM spermidine were the most efficient ones in controlling the microbial activity in the mango fruits (Figure 7). In mango, mechanical damages to the fruits resulted in development of fungal diseases during transportation and storage. *Penicillium expansum* is one of the most important fungi which result in production of blue mold and decrease consumer acceptance and shelf life. Mirdehghan and Rahimi (2016) showed that table grapes treated with polyamines had lower fungal contamination symptoms compared with the control fruits. Chitosan and spermidine were considered as a solution for managing postharvest rotting (Romanazzi et al., 2012).

## 4 CONCLUSION

Chitosan and spermidine maintained firmness, increased storage life, and delayed ripening of mango fruits and had significant positive effects on storage qualities of mango including weight, flavor, and vitamin C. Also, it was observed that by increasing the concentrations of chitosan and spermidine, total antioxidant and phenol contents of the fruits increased, that is, fruits treated with high concentrations of chitosan and spermidine had a higher acid content and lower pH value in the fruit juice compared to the control fruits. In addition, by preventing ethylene production, chitosan and spermidine coatings delayed chlorophyll decomposition and color change in fruits. In this study, it was observed that chitosan, especially 2% chitosan, and 2 mM spermidine had significant effects on improving quality and shelf life of mango. They also had considerable antifungal effects on mango fruits and it would be better to apply them during storage period of mango.

## CONFLICT OF INTEREST

The authors declare no conflict of interest.
